# Benzyl Isothiocyanate Induces Apoptosis via Reactive Oxygen Species-Initiated Mitochondrial Dysfunction and DR4 and DR5 Death Receptor Activation in Gastric Adenocarcinoma Cells

**DOI:** 10.3390/biom9120839

**Published:** 2019-12-06

**Authors:** Khin Wah Wah Han, Wah Wah Po, Uy Dong Sohn, Hyun-Jung Kim

**Affiliations:** 1 Cell Signaling and Pharmacological Activity Lab, Department of Pharmacology, College of Pharmacy, Chung-Ang University, Seoul 06974, Korea; khinwahwahhan@gmail.com (K.W.W.H.); warwarpoo@gmail.com (W.W.P.); 2 Neuropharmacology and Stem Cell Lab, Department of Pharmacology, College of Pharmacy, Chung-Ang University, Seoul 06974, Korea

**Keywords:** benzyl isothiocyanate, gastric cancer, apoptosis, reactive oxygen species, death receptors, natural product

## Abstract

Benzyl isothiocyanate (BITC) is known to inhibit the metastasis of gastric cancer cells but further studies are needed to confirm its chemotherapeutic potential against gastric cancer. In this study, we observed cell shrinkage and morphological changes in one of the gastric adenocarcinoma cell lines, the AGS cells, after BITC treatment. We performed 3-(4,5-dimethyl-2-thiazolyl)-2,5-diphenyl-2H-tetrazolium bromide (MTT) assay, a cell viability assay, and found that BITC decreased AGS cell viability. Reactive oxygen species (ROS) analyses using 2′,7′-dichlorofluorescin diacetate (DCFDA) revealed that BITC-induced cell death involved intracellular ROS production, which resulted in mitochondrial dysfunction. Additionally, cell viability was partially restored when BITC-treated AGS cells were preincubated with glutathione (GSH). Western blotting indicated that BITC regulated the expressions of the mitochondria-mediated apoptosis signaling molecules, B-cell lymphoma 2 (Bcl-2), Bcl-2-associated X protein (Bax), and cytochrome c (Cyt c). In addition, BITC increased death receptor DR5 expression, and activated the cysteine-aspartic proteases (caspases) cascade. Overall, our results showed that BITC triggers apoptosis in AGS cells via the apoptotic pathways involved in ROS-promoted mitochondrial dysfunction and death receptor activation.

## 1. Introduction

Cancer is the second-most common fatal disease after cardiovascular disease [1,2], and several studies have shown that some dietary components exert cancer-preventing effects [3,4]. Gastric cancer is the fifth most common cancer worldwide in both men and women [5]. The most common form of gastric cancer is gastric adenocarcinoma, which has a risk of relapse and metastasis even after surgery, which is the only curative treatment for gastric cancer and is supplemented with adjuvant chemotherapy and/or chemoradiation [6]. Currently, targeted molecular therapy for gastric cancers is emphasized to establish a standard chemotherapy regimen with decreased resistance and lower non-selective toxicity [7].

Benzyl isothiocyanate, BITC, is one of the isothiocyanates, which are breakdown products of glucosinolates and are found in various edible plants belonging to the Brassicaceae family, such as broccoli, cabbage, and water cress [8]. Clinical evidence from China has demonstrated that isothiocyanates can protect against gastric cancer [9]. BITC has been studied in various cancers, such as oral cancer [10], pancreatic cancer [11], brain cancer [12], melanoma [13,14], and gastric cancer [15], to evaluate its potential for cytotoxic effects on cancer cells. Additionally, BITC has been shown to have anthelmintic [16], anti-inflammatory [17], and anti-adipogenic effects [18]. Due to the large number of therapeutic advantages that have been found in various studies, BITC has gained interest as a novel therapeutic candidate for gastric cancer, with potential chemopreventive effects.

Deregulation of the apoptotic machinery is considered to be a hallmark of cancer and fixing the deregulation can contribute to cancer cell death/removal [19]. Apoptosis can be characterized by morphological features, such as cell shrinkage, nuclear chromatin condensation, and cell detachment and budding, and by biochemical hallmarks like internucleosomal DNA fragmentation and cysteine-aspartic proteases (caspases) activation [20]. It has been well documented that apoptosis is mediated by the extrinsic death receptor-regulated apoptotic pathway and the intrinsic mitochondria-involved apoptotic pathway [19,21].

In apoptosis modulation, Caspase-8 and Caspase-9 are the two main potential initiator caspases that regulate the activation of Caspase-3, which, in turn leads to the disruption of several cellular processes [22]. It has also been shown that Caspase-8 is activated by the interaction between the death effector domains of proactive Caspase-8 (Pro-Cas-8) and an adaptor protein, Fas-associated protein with death domain, which is recruited by the clustering of TNF-related apoptosis-inducing ligand (TRAIL) receptors, death receptor DR4/TRAIL-R1 and DR5/TRAIL-R2 [23]. On the contrary, proactive Caspase-9 (Pro-Cas-9) has been reported to be activated by the formation of a complex between cytochrome c (Cyt c) and apoptotic protease activating factor-1 (Apaf-1), which is initiated by the B-cell lymphoma 2 (Bcl-2)-regulated mitochondria-mediated apoptotic pathway [24].

It has been proposed that the Bcl-2-regulated apoptotic pathway can be promoted by reactive oxygen species (ROS), which are known to interrupt mitochondrial membrane integrity and cause oxidative injury [25]. As a result of mitochondrial depolarization, the levels of the anti-apoptotic protein Bcl-2, which is localized to the outer mitochondrial membrane, are dampened [13]. On the contrary, the proapoptotic effector protein Bcl-2-associated X protein (Bax) translocates from the cytosol to the mitochondria, and releases Cyt c from the mitochondrial intermembrane space into the cytosol [26].

The aim of the present study is to explore the underlying molecular mechanism of BITC-induced cell death in the human gastric adenocarcinoma cell line, AGS cells (KCLB 21739). We hypothesized that BITC is a potential candidate for the treatment of gastric cancer as it triggers apoptosis in AGS cells. We tried to find whether BITC induces AGS cell death by eliciting the ROS-initiated mitochondria-mediated pathway and the death receptor-mediated signaling pathway.

## 2. Materials and Methods

### 2.1. Materials and Reagents

BITC was purchased from Selleckchem (Houston, TX, USA) and dissolved in dimethyl sulfoxide (DMSO) to create a 10 mM stock solution. A glutathione (GSH) (G4705, Sigma-Aldrich Inc., Darmstadt, Germany) stock solution was prepared by dissolving GSH in HPLC-grade water (Fisher Scientific Korea Ltd., Gangnam-gu, Seoul, Korea), and this stock solution was diluted in the culture medium before treatment of cells to obtain the final working solution. RPMI-1640 medium was purchased from Welgene Inc. (Daegu, Korea), and fetal bovine serum (FBS) was purchased from Corning Inc. (Corning, NY, USA). Antibodies against cleaved Caspase-3 (c-Cas-3) (9664), Caspase-8 (9746), Caspase-9 (9508), poly (ADP-ribose) polymerase (PARP) (9532), X-linked inhibitor of apoptosis protein (XIAP) (14334), DR4 (42533) and DR5 (8074) were purchased from Cell Signaling Technology (Danvers, MA, USA). Goat anti-rabbit IgG-HRP and goat anti-mouse IgG-HRP antibodies were bought from Enzo Life Sciences (Farmingdale, NY, USA). Glyceraldehyde 3-phosphate dehydrogenase (GAPDH) (sc-32233), Bcl-2 (sc-492), Bax (sc-493) and Cyt c (sc-13156) were obtained from Santa Cruz Biotechnology (Santa Cruz, CA, USA).

### 2.2. Cell Culture and Morphological Observation

The human gastric cancer cell line AGS (KCLB 21739) was obtained from the Korean Cell Line Bank (Seoul, Korea). The cells were cultured in RPMI-1640 medium containing 10% FBS and 5% antibiotics, which contained 1% penicillin-streptomycin and 0.1% amphotericin B, at 37 °C in a humidified atmosphere with 5% CO_2_ and 95% air. Cells were seeded in a petri dish as a monolayer at a 1:10 ratio (cell:total media) and were passaged every time 90% confluence was reached.

To observe the changes in cell morphology, the cells were seeded in culture dishes. When 70% confluence was achieved, the cells were treated with different concentrations of BITC (i.e., 1, 5, or 10 μM) for 48 h. Then, changes in cell morphology were observed using a fluorescence microscope (Leica, Wetzlar, Germany).

### 2.3. Cell Viability Measurement (MTT Assay)

To determine cell viability regarding the apoptosis induced by BITC, a 3-(4,5-dimethyl-2-thiazolyl)-2,5-diphenyl-2H-tetrazolium bromide (MTT) assay was performed. AGS cells were seeded in 24-well plates and maintained in RPMI-1640 media supplemented with 10% FBS and 5% antibiotics. When the cells reached 70% confluence, the medium was removed by careful aspiration, and fresh culture medium containing different concentrations of BITC (i.e., 1, 5, or 10 μM) was added to evaluate dose-dependent responses and culture medium containing 5 μM BITC was added to evaluate time-dependent responses (24–72 h). After the AGS cells were treated with specific BITC doses for the desired time periods, the cells were washed twice with 1× phosphate buffered saline (1× PBS), and MTT solution (0.5 mg/mL in 1× PBS) was added to each well. After incubation for 4 h with the MTT reagent at 37 °C in the dark, the supernatant was slowly removed, and 250 μL DMSO was added. Formazan crystals were dissolved in DMSO for 30 min in the incubator, and the solution was transferred to a 96-well plate. Absorbance was measured at 570 nm [27] using a Synergy H1 Hybrid Multi-Mode microplate reader (BioTek, Winooski, VT, USA).

### 2.4. Measurement of Intracellular ROS

Intracellular ROS generation was measured through 2′,7′-dichlorofluorescin diacetate (DCFDA; Sigma-Aldrich Inc., Darmstadt, Germany) staining [28]. Briefly, the cells were seeded in a 6-well plate. After reaching 80% confluence, the cells were washed with 1× PBS and incubated with 15 μM DCFDA for 30 min to determine time-dependent ROS analysis, and 15 μM DCFDA with or without 10 µg/mL 4′,6′-diamidino-2-phenylindole (DAPI; Roche Diagnostics, Indianapolis, IN, USA) for 30 min at 37 °C in the dark to determine BITC dose-dependent ROS analysis, which was followed by another wash with 1× PBS. The cells were, then, treated with 5 μM BITC at different time points (i.e., 2.5, 4.5, and 6 h) for time-dependent ROS production analysis and cells were treated with 100 μM hydrogen peroxide (H_2_O_2_) and 1, 5, or 10 μM BITC for BITC concentration-dependent ROS analysis. Fluorescent dichlorofluorescin (DCF) was examined with a fluorescence microscope (JULI^TM^ Smart fluorescent cell analyzer, NanoEnTek Inc., Seoul, Korea) or (Leica, Wetzlar, Germany). Next, the AGS cells were collected by trypsinization and resuspended in 500 μL of 1× PBS. The collected cells were transferred to a 96-well plate, and DCF fluorescence was measured using a fluorescence microplate reader (Synergy H1 Hybrid Multi-Mode Reader; BioTek, Winooski, VT) at excitation and emission wavelengths of 485 and 535 nm, respectively, and a fix gain-100 at each time point.

### 2.5. Cell Viability Inhibition Assay

Changes in cell viability in the presence or absence of GSH were observed by measuring dye absorbance at 570 nm, as previously described. Briefly, AGS cells were incubated in a 24-well plate. After achieving 60% confluence, cells were preincubated with 1 mM GSH for 1 h. Cells were then either treated with 5 or 10 μM BITC for 48 h and control cells were incubated with DMSO and water for the same period. Cells were washed with 1× PBS and MTT reagent was, then, added for 4 h. The resulting yellow formazan crystals were dissolved in DMSO for 30 min in an incubator and the absorbance was, then, measured with a microplate reader (Synergy H1 Hybrid Multi-Mode Reader; BioTek, Winooski, VT, USA).

### 2.6. Preparation of Cellular Extracts and Western Blot Analysis

Human gastric adenocarcinoma cells were grown until 80% confluence was achieved and, then, treated with the desired concentrations of 5 or 10 μM BITC in RPMI-1640 medium containing 3% FBS for the 24 h BITC treatment; cells were treated with 1, 5, or 10 μM BITC for the 48 h treatment. After treatment, the cells were washed twice with ice-cold 1× PBS after the floating cells were collected following centrifugation at 1000 rpm for 5 min. Then, the cells were lysed using NP-40 lysis buffer [29] and harvested by scraping on ice. The lysates were incubated on ice for 30 min and vortexed four times for 10 s. Afterwards, the lysates were centrifuged at 14,000 rpm at 4 °C for 30 min. Protein quantification was carried out with a Pierce BCA protein assay kit (Pierce Biotechnology, Rockford, IL, USA). The lysates (30–50 μg) were subjected to 10–15% sodium dodecyl sulfate gel electrophoresis (SDS-PAGE) and transferred to polyvinylidene difluoride (PVDF) membranes (Merck Millipore Ltd., Tullagreen, Carrigtwohill, Co. Cork, IRL) using a Power Pac power supply (Bio-Rad, Melville, NY, USA). After blocking with 5% skim milk or 5% bovine serum albumin for 1 h, the membranes were incubated with the designated primary antibodies overnight at 4 °C at a 1:1000 dilution, with an exception of the primary antibody against Bcl-2, for which a 1:700 dilution was used. The membrane was, then, washed three times with tris-buffered saline containing 0.1% Tween 20 and incubated with secondary HRP-labeled antibodies at a 1:5000 dilution to detect the expression of the proteins of interest. Protein bands were examined using an enhanced chemiluminescence reagent (Santa Cruz Biotechnology, Santa Cruz, CA, USA) and exposed to X-ray photographic films in a dark room. Image J was used to analyze the data.

### 2.7. Statistical Analysis

All data were expressed as the mean ± the standard error of the mean (SEM) of at least three independent experiments. Statistical differences among the groups were analyzed by Student’s *t*-test. A *p*-value of 0.05 or less was considered to be statistically significant.

## 3. Results

### 3.1. BITC Inhibits AGS Cells Survival

**Figure 1:**
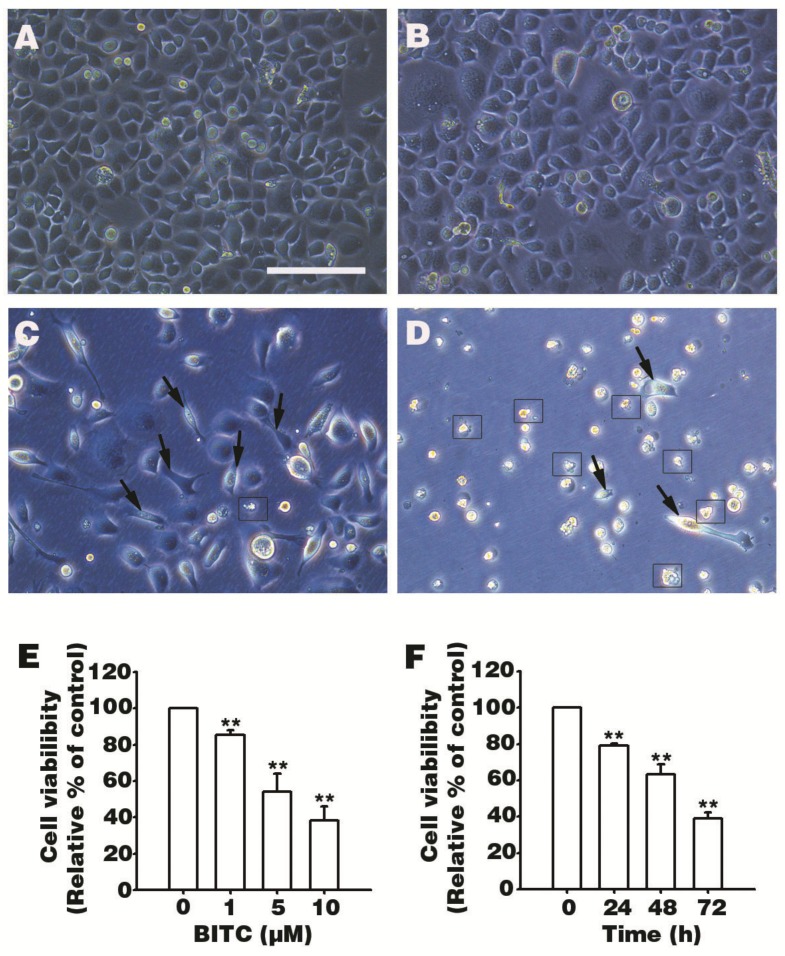
Effects of benzyl isothiocyanate (BITC) on human gastric adenocarcinoma cell line, AGS, cell survival. Cells were treated with 0.1% dimethyl sulfoxide (DMSO) (**A**) and different concentrations (1, 5, or 10 μM) of BITC for 48 h (**B**–**D**) and morphological changes were observed using a fluorescence microscope (Leica, Wetzlar, Germany) (scale bar = 100 μm). Arrows indicate cell shrinkage and squares describe membrane fragmentation. A dose-dependent cell viability assay, 3-(4,5-dimethyl-2-thiazolyl)-2,5-diphenyl-2H-tetrazolium bromide (MTT) assay, was performed after a 48 h treatment with the above mentioned doses (**E**). Next, the time-dependent effect of 5 μM BITC on AGS cells was determined at different time points (24–72 h) (**F**). Data are expressed as the mean ± SEM of three independent experiments and as the relative percentage compared to the control group. Statistical analyses were performed, and the results were compared with those of the control group. ** *p* value < 0.01.

To investigate the effects of BITC in AGS cells, we first observed AGS cells morphology after treatment with BITC. After 48 h, cells treated with 5 and 10 μM BITC presented apoptotic characteristics, such as cell shrinkage and membrane fragmentation (Figure 1A–D). Next, MTT assay was performed to evaluate viable cell levels after BITC treatment. After 48 h, the dose-dependent toxicity of BITC was observed at 1, 5, or 10 μM BITC (Figure 1E). Cell death was induced by 5 μM BITC, and 54% viable cells were observed compared to control group. Subsequently, the inhibitory effect of 5 μM BITC on AGS cell survival was determined by the MTT assay after treatment of cells for 24, 48, or 72 h. As shown in (Figure 1F), treatment with 5 μM BITC induced cell death in a time-dependent manner. These data suggest that BITC induces AGS cell death (Figure 1E,F).

### 3.2. BITC Induces Intracellular ROS Production

**Figure 2:**
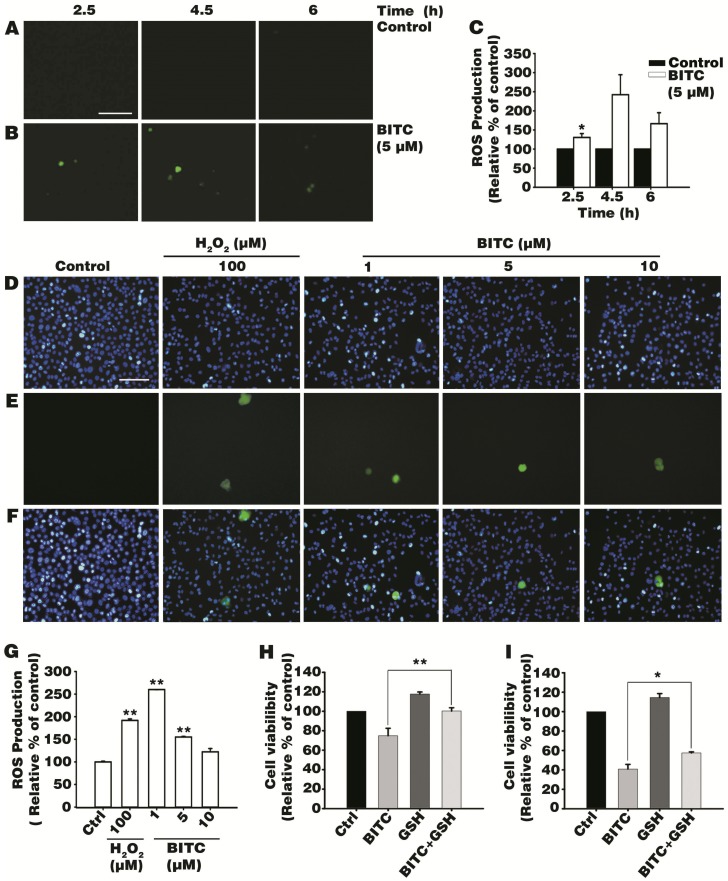
Effects of BITC on intracellular reactive oxygen species (ROS) generation and the inhibition of AGS cell death with the antioxidant glutathione (GSH). Cells were treated with 0.05% DMSO in the control group (**A**) and with 5 μM BITC in the treatment group (**B**) at 2.5 h, 4.5 h, and 6 h. After 2′,7′-dichlorofluorescin diacetate (DCFDA) staining, fluorescent DCF fluorescence was examined with a JULI^TM^ Smart fluorescent cell analyzer (scale bar = 250 μm) (**A**,**B**). (**C**) DCF fluorescence intensity in AGS cells was measured with a fluorescence microplate reader. Nuclei of cells (**D**), ROS production (**E**), and merged fluorescence (**F**) were analyzed using a fluorescence microscope (Leica, Wetzlar, Germany) by 4′,6′-diamidino-2-phenylindole (DAPI) and DCFDA staining after treatment with 0.1% DMSO, 100 μM hydrogen peroxide (H_2_O_2_) and 1, 5, or 10 μM BITC at 4.5 h (scale bar = 100 μm) (**D**–**F**). (**G**) DCF fluorescence intensity was determined with a fluorescence microplate reader. (**H**,**I**) Cells were treated with either 5 (**H**) or 10 μM BITC (**I**) for 48 h, with or without 1 mM GSH, and cell viability was measured via MTT assay. Data are expressed as mean ± SEM of three independent experiments and as the relative percentage compared to the control group. Statistical analyses were performed, and the results were compared with those of the control group. * *p* value < 0.05 and ** *p* < 0.01.

To determine how BITC induces AGS cell death, we designed an experiment to observe the ROS generated in BITC-treated AGS cells. A DCFDA assay was conducted to evaluate intracellular ROS production in AGS cells after time-dependent treatment (i.e., 0, 2.5, 4.5, or 6 h) with 0.05% DMSO and 5 μM BITC (Figure 2A,B). Abundant DCFDA positive signals indicating ROS generation were found in the BITC time-dependent treatment (Figure 2B). A peak in ROS accumulation was observed at 4.5 h after treatment with 5 μM BITC, with the relative ROS levels (242%) compared to the control group. ROS production declined at 6 h after treatment with BITC (Figure 2C). Next, BITC dose-dependent treatment was investigated at 4.5 h after AGS cells were treated with 0.1% DMSO, the positive control, H_2_O_2_ (100 μM), and different concentrations of BITC (1, 5, or 10 μM) (Figure 2D–F). The highest ROS accumulation (260%) in AGS cells was observed at the BITC low dose treatment (1 μM) (Figure 2G). At the 5 and 10 μM BITC treatment, 155% and 122% of ROS production were observed compared to the control group respectively. Taken together, these results show that BITC triggers intracellular ROS production in AGS cells.

### 3.3. Antioxidant Glutathione Ameliorated BITC-Induced AGS Cell Death

To identify the role of ROS in BITC-induced AGS cell death, we treated AGS cells with BITC in the presence or absence of the antioxidant, GSH. GSH is a commonly used antioxidant that prevents cellular damage caused by oxidative stress [30]. Treatment with GSH at physiological concentrations (1 to 10 mM) followed by treatment with apoptotic stimuli was found to repress apoptotic effects in lung epithelial cells [31]. AGS cells were pretreated with 1 mM GSH for 1 h, after which, 5 or 10 μM BITC was added and incubated for an additional 48 h. Then, 5 or 10 μM BITC-triggered AGS cell death was quantified by MTT assay (Figure 2H,I). To evaluate the hypothesis that BITC promotes ROS-induced AGS cell death, we compared the relative percentage of viable cells between the cells treated with only BITC and those treated with a combination of BITC and GSH. AGS cells treated with BITC alone resulted in 75% and 41% AGS cells survival in 5 and 10 μM BITC treatment, respectively, compared to the control group. Thus, a partial recovery from BITC-triggered cell death was observed in the cells that had been treated with both GSH and either 5 or 10 μM BITC by 28% and 16%, respectively (Figure 2H,I). These data indicate that BITC-induced cell death is mitigated by GSH and that ROS are involved in BITC-induced AGS cell death.

### 3.4. BITC Increases the Expression of DR4 and DR5 TRAIL Death Receptors

**Figure 3:**
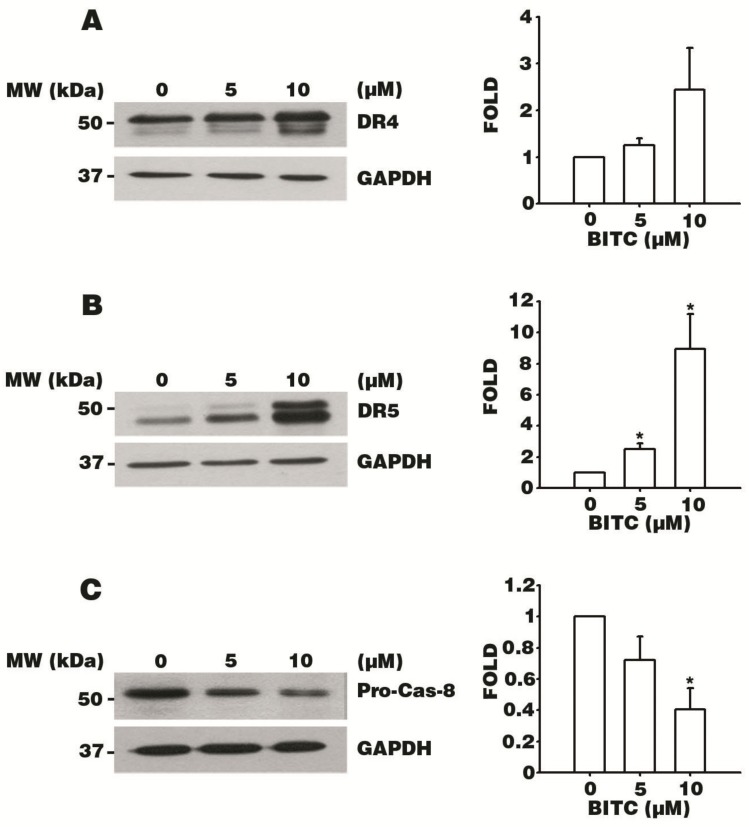
BITC induces apoptosis via the death receptor DR4/DR5-mediated pathway. AGS cells were treated with 0.1% DMSO and 5 or 10 μM BITC for 24 h, and protein lysates were extracted. The protein levels of DR4 (**A**), DR5 (**B**), and proactive Caspase-8 (Pro-Cas-8) (**C**) were analyzed by western blotting. Glyceraldehyde 3-phosphate dehydrogenase (GAPDH) was used as the loading control. Densitometer data are expressed as the mean fold change ± SEM of three independent experiments. Statistical analyses were performed by comparing the results to those of the control group. * *p* value < 0.05.

Given that cell morphologies related to apoptosis were observed upon treatment with BITC (Figure 1A–D), we hypothesized that BITC might induce AGS cell death by regulating the apoptotic machinery. The protein expressions of the DR4 and DR5 TRAIL-inducing ligand death receptors during AGS cell death were analyzed via western blotting after 5 or 10 μM BITC treatment for 24 h on AGS cells. Interestingly, the levels of both the truncated and complete isoforms of DR4 and DR5 increased in a BITC dose-dependent manner (Figure 3A,B). The higher expressions of DR4 and DR5 in BITC-treated cells compared to those in control cells imply that the death receptors were activated by BITC treatment to initiate TRAIL-induced apoptosis. We further examined Pro-Cas-8 expression and found that it remarkably decreased in a BITC dose-dependent manner, accounting for two-fifths of the 0.1% DMSO-treated control cells in the 10 μM BITC treatment (Figure 3C). The downregulation of Pro-Cas-8 indicates that the extrinsic initiator Caspase-8 was activated by the auto-proteolytic cleavage process after being recruited by the death-inducing signaling complex [23]. These data suggest that BITC triggered AGS cell death via the activation of the DR4 and DR5 TRAIL death receptors.

### 3.5. BITC Activates the Caspase Signaling Cascade

**Figure 4:**
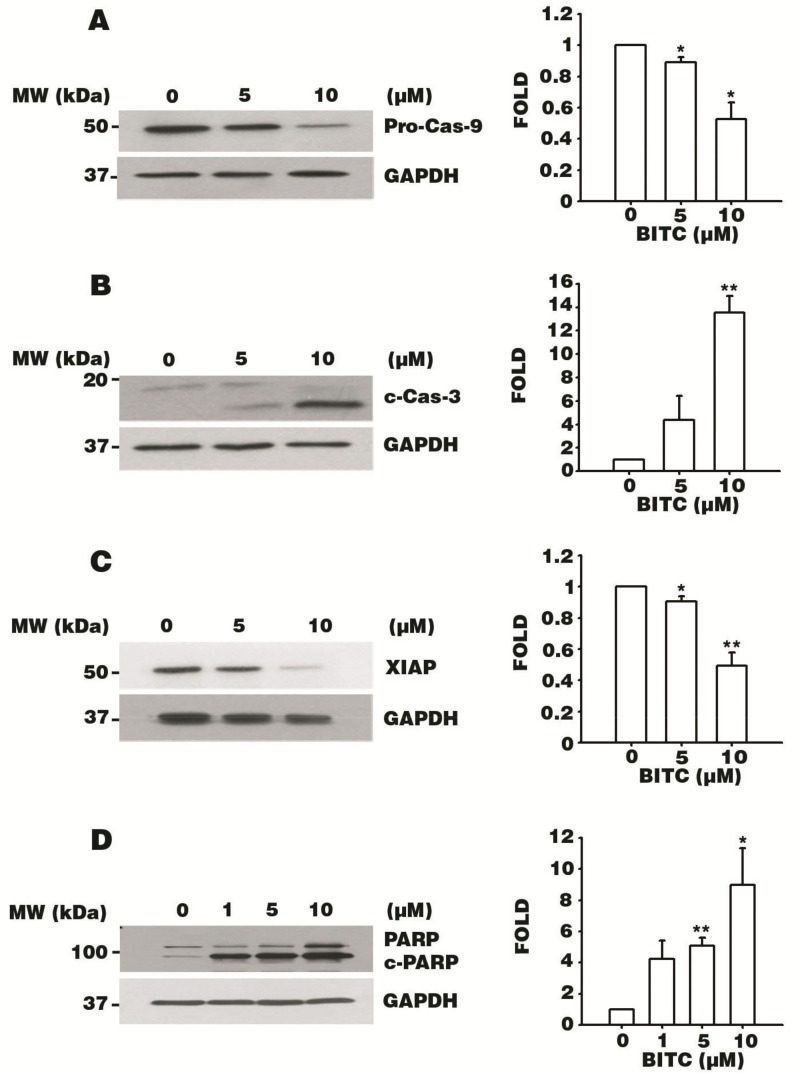
BITC induces apoptosis via the cysteine-aspartic protease (caspase)-dependent pathway. AGS cells were treated with 0.1% DMSO and 5 or 10 μM BITC for 24 h (**A**–**C**), or 1, 5, or 10 μM BITC for 48 h (**D**), and protein lysates were extracted. The expression of specific proteins was detected via western blotting. The protein levels of proactive Caspase-9 (Pro-Cas-9) (**A**), cleaved Caspase-3 (c-Cas-3) (**B**), X-linked inhibitor of apoptosis protein (XIAP) (**C**), and cleaved poly (ADP-ribose) polymerase (c-PARP)/full length PARP (PARP) (**D**) were quantified by western blotting. GAPDH was used as the loading control. Densitometer data are expressed as the mean fold change ± SEM of three independent experiments. Statistical analyses were performed, and the results were compared with those of the control group. * *p* value < 0.05 and ** *p* < 0.01.

To confirm that BITC triggers apoptosis in AGS cells, we, next, explored whether caspases are activated upon BITC treatment. The expression of Pro-Cas-9 was detected by western blotting after 5 or 10 μM BITC treatment for 24 h on AGS cells and a BITC dose-dependent downregulation was observed (Figure 4A). The Pro-Cas-9 expression level was remarkably reduced in the 10 μM BITC treatment compared to the control. The downregulation of Pro-Cas-9 protein expression correlates with the formation of an apoptosome complex (Cyt c, Apaf-1, and Caspase-9), resulting in the auto-proteolytic cleavage of the Pro-Cas-9 protein [23]. Thus, the apoptosome complex downstream signal, c-Cas-3, and XIAP were evaluated by western blot analysis after 5 or 10 μM BITC treatment for 24 h. c-Cas-3 protein expression was upregulated in a dose-dependent manner in BITC-treated cells by up to 14-fold in the 10 μM BITC treatment group, compared to that of the cells of the control group (Figure 4B). In addition, a BITC concentration-dependent decreased release of XIAP was observed (Figure 4C). The elevated expression of c-Cas-3 implies that the apoptosis signal can be transduced by BITC. The downregulation of the anti-apoptotic protein XIAP suggests that BITC promotes AGS cell apoptosis. Thus, the protein level of the downstream caspase substrate, PARP, was examined and a dose-dependent increase of cleaved PARP/full length PARP (c-PARP/PARP) was observed after 1, 5 or 10 μM BITC treatment for 48 h (Figure 4D). The increased expression of c-PARP indicates that PARP was degraded by the c-Cas-3 protein resulting in cancer cell death. These data clearly indicate that BITC triggered AGS cell death through the caspase-dependent pathway.

### 3.6. BITC Induces the Bcl-2-Regulated Apoptotic Pathway

**Figure 5:**
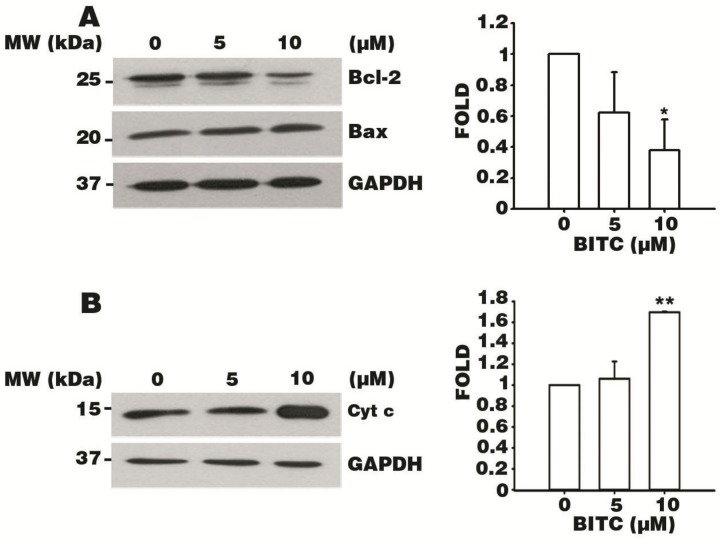
BITC induces apoptosis via the B-cell lymphoma 2 (Bcl-2)-modulated pathway. AGS cells were treated with 0.1% DMSO and 5 or 10 μM BITC for 24 h and protein lysates were extracted. Protein expressions of Bcl-2 and Bcl-2-associated X protein (Bax) (**A**) and cytochrome c (Cyt c) (**B**) were detected by western blot analysis. GAPDH was used as the loading control. Densitometer data are expressed as the mean fold change ± SEM of three independent experiments. Statistical analyses were performed by comparing the results with the data for the control group. * *p* value < 0.05 and ** *p* value < 0.01.

To further explore the molecular mechanism of apoptosis induced by BITC in AGS cells, Bcl-2-regulated apoptotic proteins were evaluated by western blotting. AGS cells were exposed to 5 or 10 μM BITC for 24 h, and the expression levels of the proteins that regulate mitochondrial function (i.e., the pro-survival marker Bcl-2 and pro-apoptotic marker Bax) were quantified. The data from the immunoblot analysis showed that there was no apparent change in Bax protein expression despite the dose-dependent decrease in Bcl-2 expression. This favored a dose-dependent reduction in the Bcl-2: Bax ratio of 10 μM BITC-treated cells of up to four-tenths of that of the control group (Figure 5A). In addition, Cyt c protein expression was found to be 1.6-fold higher in 10 μM BITC-treated cells and only slightly higher in 5 μM BITC-treated cells, compared to that of the control group (Figure 5B). These data indicate that BITC induces apoptosis of AGS cells via the Bcl-2-modulated apoptotic pathway.

## 4. Discussion

Apoptosis, or programmed cell death, is critical for the success of chemotherapies against various cancers and cancer cell resistance [32]. Failure in gastric cancer therapy may be the result of defective apoptosis process accompanied with the underlying mechanisms of Bcl-2 protein overexpression, hypoxia, and tumor microenvironment remodeling [33,34]. Nowadays, gastric cancer cells are acquiring resistance to TRAIL and various chemotherapy approaches have been investigated to increase TRAIL sensitivity in gastric cancer cells [35,36]. Thus, TRAIL, which can alter the tumor microenvironment resulting in cancer cell death [37], can be a target to reduce the chemotherapeutic resistance in gastric cancer. BITC has been reported to exert chemopreventive effects in several cancer cells by inducing anti-angiogenesis, apoptosis, cell-cycle arrest, anti-metastasis, and anti-tumorigenesis effects, and has been described to have synergistic effects with TRAIL, cisplatin, sulforaphane, phenethyl isothiocyanate, and radiation treatments [38]. BITC has been shown to inhibit AGS cell proliferation [15], but information relating to its chemotherapeutic potential in gastric adenocarcinoma has been too inconclusive to progress to its clinical use. This study was designed to explore the molecular mechanisms underlying BITC-induced AGS cell cytotoxicity.

We investigated BITC-induced ROS generation in AGS cells and its role in BITC-triggered AGS cell death. ROS generation in BITC-induced AGS cell death was examined, given that ROS production had been found to be triggered by BITC in prostate cancer cells and rat liver epithelial cells [25,39]. To the best of our knowledge, this is the first study to report that BITC-treated AGS cells produce intracellular ROS, as demonstrated by the increased number of DCF-positive cells observed upon time-dependent BITC treatment (Figure 2A,B) and dose-dependent BITC treatment (Figure 2D–F). To evaluate the impact of ROS on BITC-triggered AGS cell death, we utilized GSH to suppress BITC-induced ROS accumulation. According to our MTT assay results, AGS cell viability was restored in the presence of GSH, indicating that BITC-induced AGS cell death is truly correlated with intracellular ROS generation. Recent studies have also provided information to support the hypothesis that cytotoxic cell death is associated with intracellular ROS accumulation [40,41]. For example, apoptotic cell death has been shown to be promoted by ROS but inhibited by the antioxidant GSH [42]. Other studies that have used different cell lines have found that cell cytotoxicity ceases after treatment with GSH similarly to our data regarding the inhibition of BITC-triggered AGS cell apoptosis [11,43]. Based on these data, it is reasonable to conclude that BITC-triggered AGS cell apoptosis is promoted by ROS.

We also found that BITC modulated the expression of mitochondrial apoptosis-related proteins in AGS cells via western blot analysis. Bcl-2, a mitochondrial membrane stabilizing protein, has been shown to inhibit apoptosis by binding to pro-death proteins, such as Bax, Bcl-2-associated agonist of cell death, and Bcl-2 homologous antagonist/killer [24]. When we evaluated the levels of Bcl-2 expression, we found that Bcl-2 protein levels decreased significantly after treatment with BITC, although Bax was not affected. Concomitantly, the release of Cyt c was observed with the dose-dependent increase in protein expression compared to that in the control group. According to recent studies, Bcl-2 was found to be downregulated while Bax was upregulated in the presence of an apoptotic signal, and Cyt c was observed to be released as a result of altered mitochondrial membrane permeability [13,44]. Bcl-2 was shown to phosphorylate without the expression alteration of Bax levels in human bladder cancer cells, which is similar to our study that Bax level was not changed by BITC [45]. It has further been suggested that this discrepancy in the modulation of the Bcl-2 associated proteins by isothiocyanates may be correlated with the concentrations of the treatments. However, an accepted explanation for the dissociation of Bcl-2 family proteins in BITC-triggered apoptosis remains unknown. The results of the previous study [45] may explain why we found that the Bax expression level did not change upon exposure to BITC in AGS cells, while a downregulation of the Bcl-2 expression level was observed. Our data demonstrate that BITC favors a dose-dependent decrease in the Bcl-2: Bax ratio and the release of Cyt c in AGS cells. Therefore, we hypothesize that BITC triggers AGS cell death by initiating the Bcl-2-regulated apoptotic signaling pathway.

The regulation of the expression of the proactive form of the caspase initiator of the intrinsic apoptotic pathway, Pro-Cas-9, may be one of the important checkpoints of mitochondrial dysfunction in BITC-triggered apoptosis. When we evaluated Pro-Cas-9 expression after BITC treatment, we found that it was downregulated. On the contrary, the expression of XIAP, which may inhibit the cleavage of the Caspase-9 initiator and the Caspase-3 executor [46,47], was reduced in a dose-dependent manner with the reduction in the levels of Pro-Cas-9 expression and elevation in the levels of c-Cas-3 expression. This hyperactivation of the caspases has been found to contribute to membrane blebbing in apoptotic cells [23]. Similar results can be found in a previous study [45] that supports the premise that BITC-induced mitochondrial damage is involved in the apoptotic machinery. The data produced in this study clearly suggest that mitochondrial dysfunction plays a role in BITC-triggered AGS cell apoptosis.

Caspases activation may be mediated by an another pathway, the death-receptor-mediated apoptotic pathway [48], and the elevated expressions of DR4 and DR5 have been found to be correlated with the cellular sensitivity to TRAIL-mediated apoptosis [49]. In the current study, we showed that BITC initiates caspases activation via the extrinsic apoptotic pathway by regulating DR4, DR5, and Caspase-8. When we observed membrane-bound TRAIL receptors activation during BITC-induced apoptosis, we found that BITC upregulated both DR4 and DR5 expressions. This hyperactivation of the death receptors initiated the caspases cascade. PARP, a downstream caspase substrate, can be cleaved by c-Cas-3, which results in the disruption of several cellular processes and leads to apoptosis [50]. As such, BITC-induced AGS cell death was triggered by the DR4- and DR5-modulated apoptotic pathway. This result is supported by previous studies that poncirin and tangeretin also induced apoptosis through the death receptor-mediated apoptotic pathway [51,52]. Other studies have reported similar results that show that the molecular mechanisms of apoptosis in gastric cancer cells are related to DR4 [53] and DR5 [54]. These data support the hypothesis that the regulation of DR4 and DR5 is a key molecular mechanism underlying apoptosis in AGS cells.

Interestingly, a study on glioma cells found that the apoptosis signaling cascade was initiated by the specific death receptor DR5, but not DR4 [55]. However, in human cancer stem-like cells, upregulation of both DR4 and DR5 was found to contribute to apoptosis [56]. Therefore, death receptor activation that is initiated by a cell death stimulus appears to be specific for particular cell types [57]. On the contrary, DR4 is considered to play a pivotal role in apoptosis and its dysfunction can promote tumor cell metastasis [58]. Hence, the upregulation of both DR4 and DR5 in BITC-induced AGS cell death may be advantageous for enhancing the responsiveness of AGS cells to TRAIL ligands.

Overall, our findings highlight the importance of the apoptotic machinery in BITC-triggered AGS cell death. Moreover, our results provide evidence that BITC provokes DR4/DR5-dependent and mitochondria-mediated apoptosis, which is accompanied by the intracellular generation of ROS. These conclusions are supported by a recent study that used different cell lines to determine the involvement of extrinsic and intrinsic apoptotic pathways in BITC-induced cell death [13]. These new findings greatly improve our understanding of the molecular mechanisms of BITC-induced AGS cell death and pave the way for designing novel chemotherapeutic strategies for gastric cancer. However, the link between the different signaling pathways that promote BITC-triggered AGS cell death requires further investigation. Previous studies showing that chemotherapeutic efficacy can be obtained with BITC doses below the genotoxic levels, both in vitro and in vivo [59], and negligible or absence of BITC toxicity in normal PBMCs [60], normal breast MCF-10A cells [61,62], and normal HPDE-6 cells [63] indicate that BITC could be a novel chemotherapeutic agent with low toxicity in normal cells. According to our data, BITC could be helpful to gastric cancer patients because it induces apoptosis in gastric cancer cells. As BITC is a dietary phytochemical, it can be easily supplemented as a diet or pill. BITC may enhance the effects of chemotherapies in gastric cancer patients when it is given in combination with chemotherapies or it may be helpful for gastric cancer prevention when it is ingested daily as a food supplement.

## 5. Conclusions

**Figure 6:**
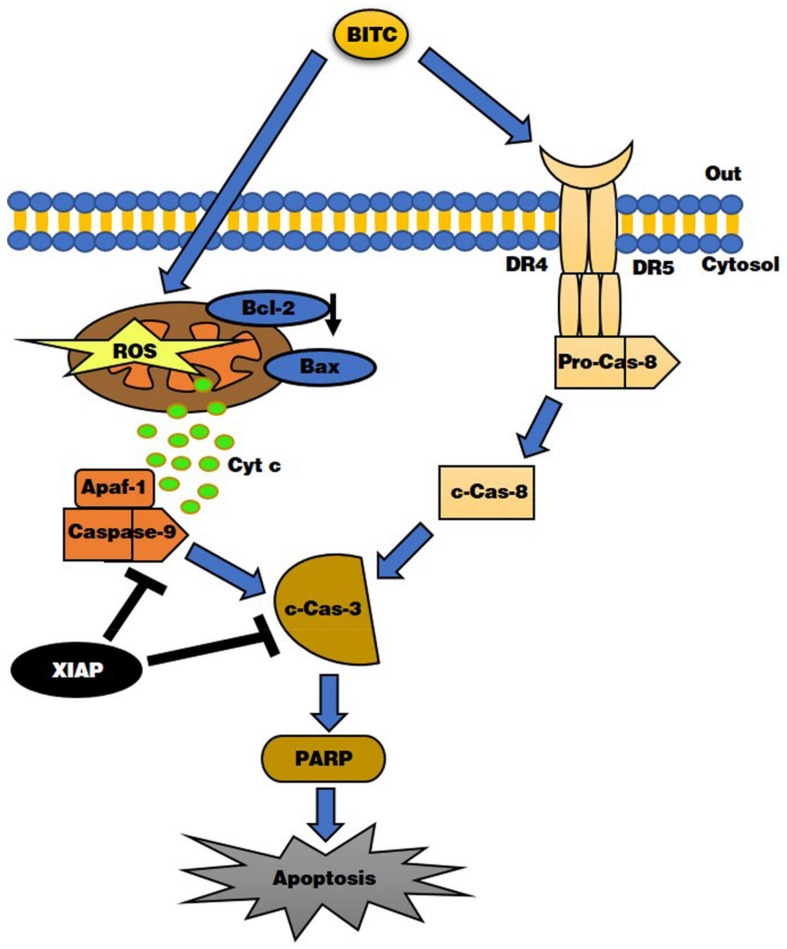
Proposed model of the signaling pathways underlying BITC-induced. BITC induced apoptosis in AGS cells via the death receptor-mediated apoptotic pathway and the mitochondria-mediated apoptotic pathway.

The present study demonstrated that BITC-triggered AGS cell death is mediated by apoptotic pathways. Our data showed that BITC-induced AGS cell death is carried out via the DR4/DR5-regulated caspase-dependent pathway and the mitochondrial dysfunction mediated by the ROS production. To the best of our knowledge, this study is the first to show that BITC induces AGS cell death via apoptosis signaling pathways. A proposed model depicting the molecular mechanism underlying BITC involvement in AGS cell death is presented in Figure 6. In accordance with our data, BITC could be used to reduce the occurrence of or treat cancer in gastric cancer patients in the future. In summary, our findings highlight that BITC may be promising as a candidate for the development of gastric cancer chemotherapies or as a potential food supplement to lessen the occurrence of gastric cancer worldwide.
